# Development and implementation of a TaqMan triplex real-time PCR assay for concurrent detection of pseudorabies virus, porcine teschovirus 1, and *Streptococcus suis 2*

**DOI:** 10.3389/fvets.2025.1589175

**Published:** 2025-06-18

**Authors:** Ranran Lai, Chen Yang, Lili Wu, Weisheng Wu, Lulu Li, Wei Liu, Zheng Yan, Diankun Yu, Shengzhi Ren, Zhiqiang Hu, Xiaowen Li

**Affiliations:** ^1^Shandong Engineering Research Center of Pig and Poultry Health Breeding and Important Infectious Disease Purification, Shandong New Hope Liuhe Group Co., Ltd., Qingdao, China; ^2^Juye Xinhao Agriculture and Animal Husbandry Co., Ltd., Heze, China; ^3^China Agriculture Research System-Yangling Comprehensive Test Station, Yangling, China; ^4^Key Laboratory of Feed and Livestock and Poultry Products Quality & Safety Control, Ministry of Agriculture, New Hope Liuhe Co., Ltd., Chengdu, China; ^5^Key Laboratory of Animal Epidemic Disease Detection and Prevention in Panxi District, College of Animal Science, Xichang University, Xichang, China

**Keywords:** triplex real-time PCR, porcine nervous diseases, PRV, PTV1, SS2

## Abstract

**Introduction:**

Porcine neurological disorders represent a prevalent clinical condition that leads to significant mortality and economic losses within the swine industry. Pseudorabies virus (PRV), porcine teschovirus 1 (PTV1), and *Streptococcus suis 2* (SS2) are key viral and bacterial pathogens implicated in the manifestation of neurological symptoms in pig populations. The overlapping clinical presentations and pathological alterations associated with these pathogens pose challenges in their clinical differentiation. Therefore, it is essential to develop a diagnostic method with high sensitivity and specificity that can simultaneously detect and differentiate these viral and bacterial agents.

**Materials and methods:**

A triplex real-time PCR assay using TaqMan probes was developed to simultaneously detect PRV, PTV1, and SS2. To assess the efficacy of the established assay, 30 clinical samples of animals with nervous symptoms were used to compare the results obtained from the triplex real-time PCR assay with those obtained from commercial singleplex real-time PCR kits. Furthermore, a total of 282 samples were tested and analyzed to validate the utility of the assay.

**Results:**

The triplex real-time PCR assay exhibited high sensitivity, specificity, and repeatability, with a detection limit of 1.0 × 10^0^ copy/μL. The triplex real-time PCR method and commercial singleplex real-time PCR kits showed complete concordance in detecting PRV, PTV1, and SS2. Clinical data indicated single infection rates of 8.16% for PRV, 26.95% for PTV1, and 7.80% for SS2. The observed co-infection rates were 7.45% for PRV + PTV1, 0.71% for PRV + SS2, 1.42% for PTV1 + SS2, and 1.77% for PRV + PTV1 + SS2, respectively.

**Conclusion:**

The triplex real-time PCR method developed in this study effectively distinguishes PRV, PTV1, and SS2 simultaneously, serving as a valuable diagnostic tool. This method is anticipated to play a crucial role in preventing and controlling infectious disease spread and supporting epidemiological investigations.

## 1 Introduction

Neurological diseases significantly threaten the swine industry, leading to considerable economic losses worldwide (1). Swine are affected by various neurological diseases such as edema disease (ED), pseudorabies virus (PRV), porcine teschovirus (PTV), encephalomyocarditis virus (EMCV), *Streptococcus suis* (SS), and *Haemophilus parasuis* (HPS) (2–6). Among these swine neurological diseases, PRV, PTV, and SS are the most destructive pathogens with high infection rates and mortality rates, causing anorexia, spasms, seizures and miscarriages.

Pseudorabies, caused by the PRV, is a neurotropic disease that causes significant neurological dysfunction in affected animals. This globally prevalent disease poses considerable challenges for eradication. It is a double-stranded linear DNA virus belonging to the herpesvirus family type 1. Its genome is approximately 150 Kb, encoding 70–100 viral proteins, including pivotal glycoproteins like gE and TK that affect its pathogenicity. Various mammalian species, including humans, have been identified as potential hosts for this pathogen (7–9). In pigs, it causes severe neurological symptoms with nearly 100% mortality in piglets less than 2 weeks old, reproductive issues in sows, and reduced breeding value in boars. PRV spreads quickly year-round through latent infections and ongoing viral shedding, hindering eradication and causing persistent infections (10). Before 2011, China effectively controlled pseudorabies with the Bartha K61 vaccine, but new variant strains later led to significant losses in the swine industry (8, 11).

PTV is a positive-stranded RNA virus classified under the *Teschovirus* genus of the *Picornaviridae* family. The first reported case of PTV occurred in Czechoslovakia in 1929, followed by subsequent outbreaks in many countries (12–14). The PTV genome is approximately 7.2 kb in length, and its vision is non-enveloped and consists of an internal core and a protein capsid with a diameter of about 20–30 nm (15). There are 13 serotypes, including 12 serotypes (PTV1 ~ PTV12) detected in domestic pigs and a PTV13 serotype detected in wild boar (16). Clinically, PTV is primarily transmitted through the fecal-oral route within pig herds. Pigs are the only known host for PTV, and the virus typically causes subclinical infections, especially in young pigs. The mild-pathogenic strain of PTV1 and other serotypes can lead to a mild form of encephalomyelitis from which most pigs fully recover without long-term complications. However, infection with the strong-pathogenic strain of PTV1 can trigger an epidemic outbreak manifesting typical neurological symptoms along with diarrhea, encephalomyelitis, reproductive disorders in sows, and myocarditis (17–19). The World Organization for Animal Health (WOAH) classifies the highly lethal encephalomyelitis caused by the virulent PTV1 strain as a B-class contagious disease, given its mortality rate of 80% or more, causing significant negative impacts on social and economic aspects that should not be underestimated. Co-infections involving PTV1 and other pathogens frequently occur in clinical settings, complicating disease presentation and diagnostic processes in veterinary clinics (20–23).

Swine streptococcosis is an important zoonotic infectious disease caused by various strains of SS, which was first reported in the Netherlands in the 1960s and has since spread to various countries. The WOAH has classified the disease as a B-class animal disease, and China has classified it as a type II infectious disease (24). SS is a gram-positive facultative anaerobic coccus, with a complex and diverse array of serotypes due to differences in capsule polysaccharide antigens (CPS), which can be divided into 35 serotypes. Specially, SS2 is the most widespread and virulent serotype, displaying strong survival capabilities in complex environments (25–28). SS2 is primarily transmitted through the mouth or respiratory tract. When infecting humans and animals, it has the characteristics of sudden onset, high infection rate, and irreversible sequelae (29, 30). Pigs infected with SS2 may exhibit symptoms such as fever, sepsis, lymphadenitis, and meningitis. Recent reports of human SS2 infections have increased dramatically, with infected individuals showing symptoms such as meningitis, arthritis, and sepsis that can lead to death (24, 31–33). Therefore, SS2 poses a potential threat to the global swine industry, public health, and food safety in various countries around the world, and should be given high priority.

Pigs infected with PRV, PTV1, and SS2 show indistinguishable clinical symptoms and pathological changes, complicating their differentiation. The occurrence of co-infections and secondary infections involving PRV, PTV1, and SS2 presents notable challenges in clinical diagnostics (34–36). Consequently, developing an efficient method for clinical differentiation of these diseases is urgently needed. Real-time PCR tracks the PCR process using fluorescence signals, providing faster, more sensitive, and reproducible results than traditional PCR techniques. Therefore, it is frequently utilized in clinical diagnostics. Singleplex real-time PCR is inefficient for detecting multiple pathogen co-infections simultaneously, as it requires repeated testing, which is time-consuming and complicates procedures (37, 38). Multiplex real-time PCR enables the simultaneous detection of multiple pathogens within a single reaction system (39, 40). This study presents a TaqMan probe-based triplex real-time PCR method for the simultaneous and precise detection of PRV, PTV1, and SS2. The assay demonstrated high sensitivity and specificity for the target genes. We applied this method to analyze 282 clinical samples from pig farms, offering valuable data for developing prevention and control strategies in the region.

## 2 Materials and methods

### 2.1 Viruses, primers, and probes

The nucleic acid of various viruses and bacteria, including African swine fever virus (ASFV), porcine reproductive and respiratory syndrome virus (PRRSV), porcine epidemic diarrhea virus (PEDV), porcine circovirus type 2 (PCV2), porcine circovirus type 3 (PCV3), classical swine fever virus (CSFV), transmissible gastroenteritis virus (TGEV), PRV, PTV1, and SS2 were stored at −80°C in our laboratory. 85 genome sequences of PRV, 18 genome sequences of PTV1 and 18 genome sequences of SS2 were downloaded from NCBI and analyzed using MEGA software (Version 11.0). After comparison, the gE gene of PRV, the VP1 gene of PTV1, and the CPS2J gene of SS2 were found to be the most conserved gene sequences and selected to be target genes. Primers and probes were designed with Primer Premier 5 software (Premier, Canada) targeting the most conserved region. TaqMan probes for PRV, PTV1, and SS2 were labeled with FAM, VIC, and Cy5 at the 5′ end, respectively, with all quenchers at the 3’end being BHQ. Table 1 displays the sequences of the primers and probes developed in this study. Sangon Biotech (Shanghai) Co., Ltd. synthesized the primers and probes.

**Table 1:** Primers and probes designed for the triplex real-time PCR.

Virus	Primer/Probe	Sequence (5′-3′)	Size (bp)	Target gene
PRV	Forward	GCTTCCACGCGCTCGGCTTC	188	gE
	Reverse	CGGGTGGTAGATGCAGGGCT		
	Probe	(FAM)CGACCTGATGCCGCGCGTGGTCT(BHQ1)		
PTV1	Forward	CCTTCTGAAAGACCTGCTCTG	114	VP1
	Reverse	CAGTCCCATTGCCCAGTC		
	Probe	(VIC)CTTCTGTACCCTGTCGCCACCATTG(BHQ1)		
SS2	Forward	GATAGATGACGGTTCTTCAGATTC	93	CPS2J
	Reverse	CCATTTGGTAACCGGAAAAGTT		
	Probe	(CY5)TCTACCATCTTGCTCTGCGTATTCCA(BHQ2)		

### 2.2 Construction of plasmid standards

The target fragments of PRV-gE, PTV1-VP1, and SS-CPS2J were amplified separately using the HiScript II One Step RT-PCR Kit (Dye Plus) (Cat: P612, Nanjing Vazyme Biotech Co., Ltd., Nanjing, China) and the corresponding primers listed in Table 1. The PCR fragments were purified and inserted into the pMD18-T vector (Cat: 6011, Takara Biomedical Technology, Beijing, China). The *E.coli* DH5α strain was transformed with the clones. Positive clones were cultured, and plasmid extraction was conducted using the TaKaRa MiniBEST Universal Genomic DNA Extraction Kit (Cat: 9765, Takara Biomedical Technology, Beijing, China). DNA sequencing verified the constructed plasmid, which served as the standard positive control. The recombination plasmid copy number was determined using the formula outlined in reference (41):


$$\text{Plasmid}\;\;\text{copies}/\mathrm{\mu L}=\frac{\left(6.02\times 10^{23})\times (X\;\;\mathrm{ng}/\mathrm{\mu L}\times 10^{-9}\right)}{\text{plasmid}\;\;\text{length}(\mathrm{bp})\times 660}$$

Each plasmid underwent a tenfold serial dilution, spanning from 1.0 × 10^9^ copies/μL to 1.0 × 10^0^ copies/μL. Singleplex real-time PCR was conducted for each virus with 10-fold diluted plasmids to create a standard curve. Each plasmid was diluted to 3.0 × 10^9^ copies/μL and combined in equal volumes to achieve a concentration of 1.0 × 10^9^ copies/μL per plasmid for mutiplex standard curves. The pooled plasmid was serially diluted tenfold from 1.0 × 10^9^ to 1.0 × 10^0^ copies/μL to create multiplex standard curves.

### 2.3 Reaction conditions for singleplex real-time PCR

Each real-time PCR reaction was conducted in a 20 μL volume. The singleplex real-time PCR for PRV, PTV1, and SS2 was conducted using a reaction system comprising 10 μL of 2 × One Step Q Probe Mix, 1 μL of One Step Q Probe Enzyme Mix (Cat: Q222, Nanjing Vazyme Biotech Co., Ltd., Nanjing, China), 0.6 μL each of forward and reverse primers (10 μM), 0.3 μL of TaqMan probe (10 μM), 4 μL of template, and 3.5 μL of nuclease-free water. The amplification process utilized a Bio-Rad CFX96™ Real-time System (Bio-Rad, Hercules, CA, United States) with the following protocol: initial steps at 55°C for 5 min and 95°C for 30 s, followed by 40 cycles of 95°C for 10 s and 60°C for 30 s. Fluorescence signals were automatically recorded at the conclusion of each cycle. All qPCR results were analyzed with CFX Manager™ software.

### 2.4 Optimized conditions of triplex real-time PCR assay

Primer and probe concentrations were optimized following the method outlined in the reference (42). The optimal conditions for triplex real-time PCR were established as: 10 μL 2 × One Step Q Probe Mix, 1 μL One Step Q Probe Enzyme Mix (Cat: Q222, Nanjing Vazyme Biotech Co., Ltd., Nanjing, China), 0.2 μL of each forward/reverse primer (10 μM), 0.1 μL of each probe (10 μM) for PRV, PTV1, and SS2, 4 μL of template, and 3.5 μL of nuclease-free water, totaling a reaction volume of 20 μL. The previously mentioned instrument and real-time PCR program were utilized.

### 2.5 Evaluation of the triplex real-time PCR assay focused on its sensitivity, specificity, and repeatability

The limit of detection (LOD) for the triplex real-time PCR method was assessed by performing tenfold serial dilutions of the pooled standard plasmids, from 1.0 × 10^9^ to 1.0 × 10^0^ copies/μL in nuclease-free water. The diluted standard plasmids served as templates for multiplex real-time PCR amplification. The reliable LOD was the lowest concentration that achieved a 95% positive detection rate.

To prevent false positives from other viruses in the samples, specificity tests for the triplex real-time PCR assay included four RNA viruses (PRRSV, PEDV, CSFV, and TGEV) and three DNA viruses (ASFV, PCV2, and PCV3). Nucleic acids were isolated with the VAMNE Virus DNA/RNA Extraction Kit (Cat: RM502, Nanjing Vazyme Biotech Co., Ltd., Nanjing, China). DNA and RNA samples were initially tested using commercial kits. Samples with CT values below 25 were chosen as templates for the specificity assessment of the triplex real-time PCR assay. The standard plasmids of PRV, PTV1 and SS2 were used as positive controls, while nuclease-free water served as the negative control.

The repeatability of the triplex real-time PCR was evaluated using a tenfold serial dilution of the standard template, spanning from 1.0 × 10^6^ to 1.0 × 10^2^ copies/μL. Each reaction was conducted in triplicate. Intra-assay repeatability was assessed by performing three concurrent detections of each plasmid under the same conditions. Inter-assay repeatability was assessed by conducting the assays three times at separate intervals. The coefficient of variation (CV) of the Cq values across the three experiments was computed to assess repeatability.

### 2.6 Validation of the triplex real-time PCR assay

Thirty clinical samples of animals with nervous symptoms were simultaneously detected using both the developed triplex real-time PCR method and commercial singleplex real-time PCR kits. Detection results from our methods were compared with those from singleplex real-time PCR.

### 2.7 Application of triplex real-time PCR in clinical settings

The triplex real-time PCR assay was employed to investigate the detection rates of PRV, PTV1, and SS2 across 282 samples of animals with nervous symptoms, comprising 75 fecal samples, 72 rectal swabs, 69 oral fluid samples, and 66 nasopharyngeal swabs. Samples were collected from pig farms across various provinces in China, including Shandong, Hebei, Liaoning, Shaanxi, Hunan, Hubei, Zhejiang, and Sichuan, between November 2022 and June 2024. Clinical samples were processed with phosphate buffer saline (PBS), followed by vortexing and centrifugation to collect the supernatant. Nucleic acids were isolated with the DNA/RNA Extraction Kit from Nanjing Vazyme Biotech Co., Ltd. The constructed plasmid served as the positive control, while nuclease-free water was used as the negative control. Detection rates were analyzed following the acquisition of assay results for all clinical samples.

## 3 Results

### 3.1 Individual pathogen detection using the single real-time PCR assay

**Figure 1:**
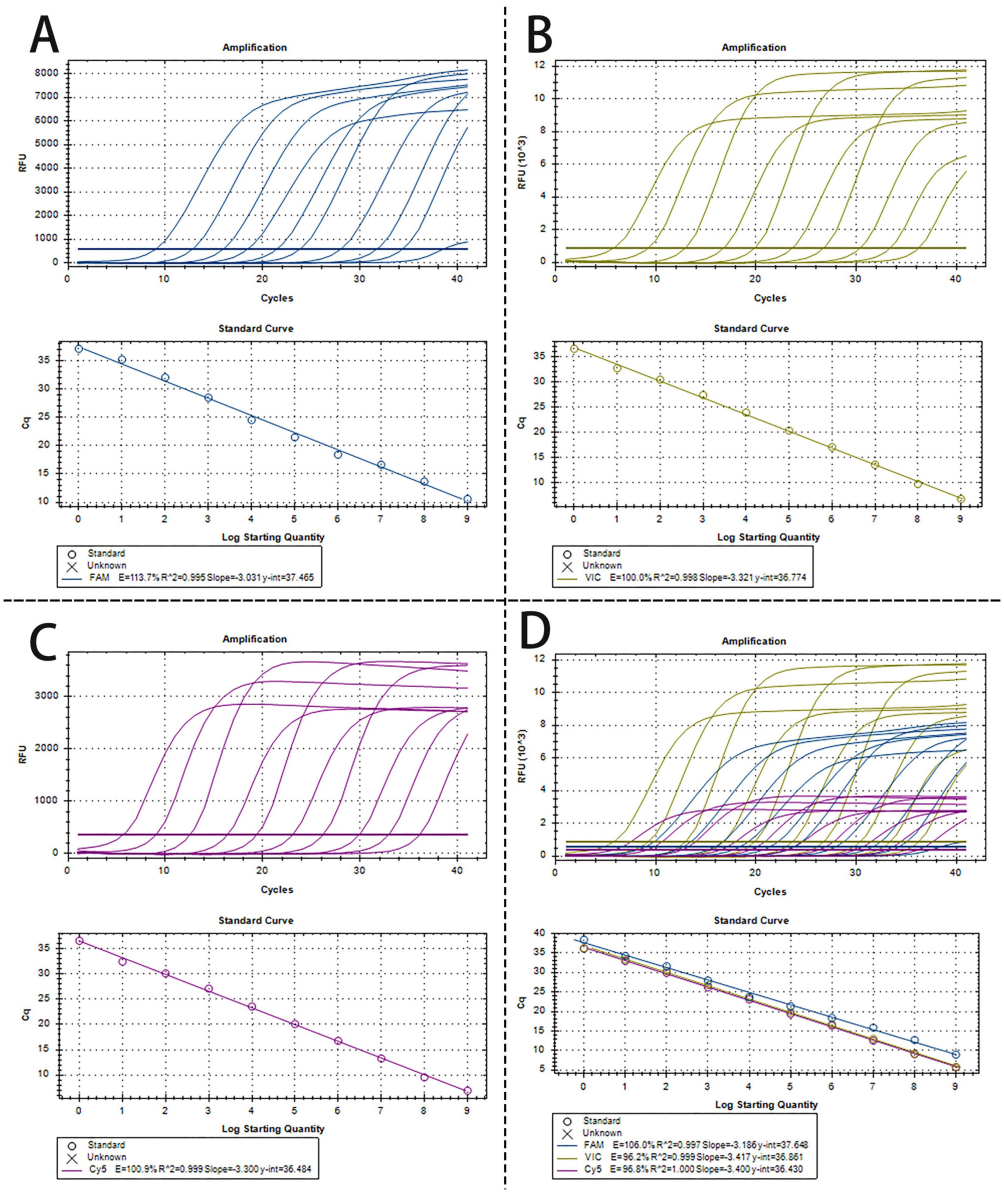
The amplification curves and the standard curve of the single and triplex real-time PCR assay. **(A–C)** The amplification curves and the standard curve (bottom) of single real-time PCR assay for detection of PRV **(A)**, PTV1 **(B)**, and SS2 **(C)**, respectively, with concentrations of each plasmid standard ranging from 1.0 × 10^9^ copies/μL to 1.0 × 10^0^copies/μL. **(D)** Amplification curves and standard curves of optimized triplex real-time PCR for simultaneous detection of PRV, PTV1 and SS2. The concentrations of each plasmid standard were from 1.0 × 10^9^ copies/μL to 1.0 × 10^0^ copies/μL.

To create a triplex real-time PCR, we initially developed singleplex real-time PCR assays for each pathogen, employing distinct fluorescence-labeled target probes. A standard curve for each pathogen was established using 10-fold serial dilutions of plasmids, spanning from 1.0 × 10^9^ to 1.0 × 10^0^ copies/μL. As shown in Figures 1A–C, all the standard curves showed excellent coefficients of determination (R^2^) and amplification efficacy (E), with PRV (R^2^ = 0.995; E = 113.7%), PTV1 (R^2^ = 0.998; E = 100.0%), SS2 (R^2^ = 0.999; E = 100.9%), respectively. This indicates that our plasmid standards were qualified, and the designed primers and probes were efficient.

### 3.2 Development of a triplex real-time PCR assay

The optimized triplex assay was tested with serial dilutions of mixed standard plasmids. The triplex real-time PCR effectively identified all target genes of the three pathogens (Figure 1D). As shown in Figure 1D, the standard curves demonstrated high R^2^ and E values for each pathogen: PRV (R^2^ = 0.997, E = 106.0%), PTV1 (R^2^ = 0.999, E = 96.2%), and SS2 (R^2^ = 1.000, E = 96.8%), confirming the validity and reliability of the triplex real-time PCR.

### 3.3 The triplex real-time PCR assay’s specificity

**Figure 2:**
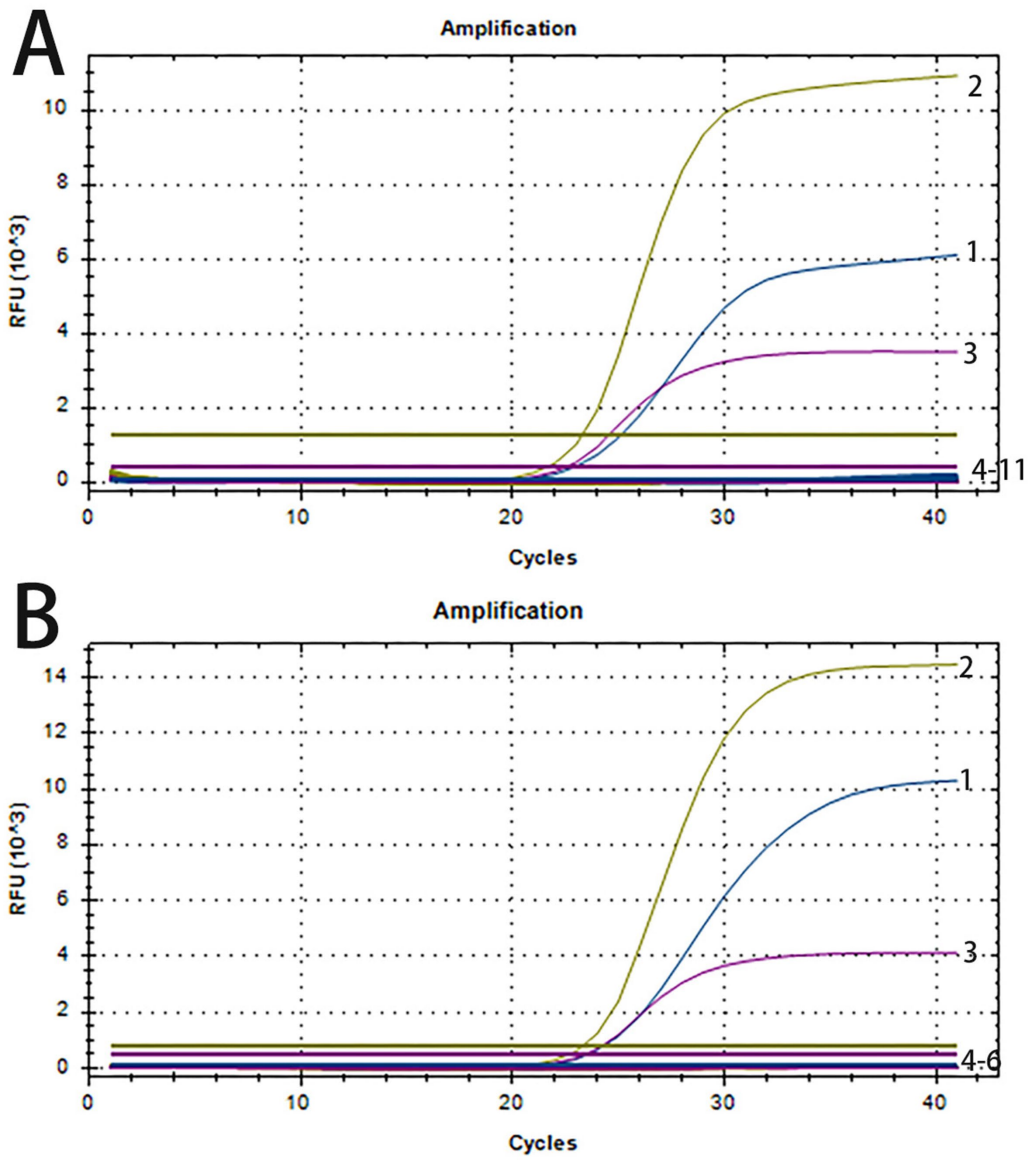
The amplification curves of specificity tests of triplex real-time PCR. **(A)** Line 1–3: positive templates of PRV, PTV1 and SS2. Line 4–11: negative control and positive templates of ASFV, PCV2, PCV3, PRRSV, PEDV, CSFV, TGEV. **(B)** 1–3: positive templates of PRV, PTV1 and SS2. 4–6: templates of clinical samples from healthy pigs.

The specificity of the triplex real-time PCR was assessed using nucleic acids of ASFV, PRRSV, PEDV, PCV2, PCV3, CSFV, and TGEV as amplification templates. The standard plasmids of PRV, PTV1 and SS2 were tested as the positive control, while nuclease-free water as negative control. Figure 2A demonstrates successful detection of all standard plasmids of PRV, PTV1 and SS2, with no positive signals from the seven other viruses or the negative control. To confirm specificity, three clinical samples from healthy pigs were further tested. Amplification curves indicated that only target genes from standard plasmids were detected, while clinical samples from healthy pigs showed no positive signals (Figure 2B). The results demonstrated the high specificity of the triplex real-time PCR assay.

### 3.4 The triplex real-time PCR assay’s sensitivity

The sensitivity of the triplex real-time PCR assay was evaluated using pooled standard plasmids at concentrations from 1.0 × 10^2^ to 1.0 × 10^0^ copies/μL under optimized conditions. Table 2 showed detection rates for all samples were more than 95% of replicates. Therefore, the reliable LOD of this method is 1 × 10^0^ copies/μL for PRV, PTV1 and SS2.

**Table 2:** The sensitivity tests of triplex real-time PCR.

Templates	Concentrations (copies/μL)	The number of tested samples	Positive number	Positive rate (%)	95% detection rate (%)
PRV	1 × 10^2^	30	30	100	> 95
	1 × 10^1^	30	30	100	> 95
	1 × 10^0^	30	29	96.7	> 95
	Negative control	30	0	0	< 95
PTV1	1 × 10^2^	30	30	100	> 95
	1 × 10^1^	30	30	100	> 95
	1 × 10^0^	30	30	100	> 95
	Negative control	30	0	0	< 95
SS2	1 × 10^2^	30	30	100	> 95
	1 × 10^1^	30	30	100	> 95
	1 × 10^0^	30	30	100	> 95
	Negative control	30	0	0	< 95

### 3.5 The triplex real-time PCR assay’s repeatability

The repeatability of the triplex real-time PCR assay was validated using standard plasmids at concentrations from 1.0 × 10^6^ to 1.0 × 10^2^ copies/μL. Table 3 indicated that CVs for Cq values ranged from 0.05 to 0.63% in intra-group tests and from 0.04 to 1.12% in inter-group reproducibility tests. These findings demonstrated that the developed triplex real-time PCR assay exhibited satisfactory repeatability.

**Table 3:** The repeatability tests of triplex real-time PCR.

Templates	Concentrations (copies/μL)	Intra-assay		Inter-assay	
		Cq values (mean ± SD)	CV%	Cq values (mean ± SD)	CV%
PRV	1 × 10^6^	18.26 ± 0.09	0.49	18.31 ± 0.06	0.33
	1 × 10^5^	21.23 ± 0.10	0.47	21.26 ± 0.11	0.52
	1 × 10^4^	23.76 ± 0.02	0.08	23.74 ± 0.01	0.04
	1 × 10^3^	27.78 ± 0.15	0.54	27.86 ± 0.11	0.39
	1 × 10^2^	31.80 ± 0.13	0.41	31.76 ± 0.14	0.44
	Negative control	ND	ND	ND	ND
PTV1	1 × 10^6^	17.17 ± 0.08	0.47	17.21 ± 0.07	0.41
	1 × 10^5^	20.36 ± 0.03	0.15	20.51 ± 0.19	0.93
	1 × 10^4^	24.01 ± 0.13	0.54	23.95 ± 0.16	0.67
	1 × 10^3^	27.29 ± 0.04	0.15	27.27 ± 0.02	0.07
	1 × 10^2^	30.84 ± 0.11	0.36	30.87 ± 0.12	0.39
	Negative control	ND	ND	ND	ND
SS	1 × 10^6^	17.13 ± 0.09	0.53	17.23 ± 0.13	0.75
	1 × 10^5^	20.24 ± 0.01	0.05	20.48 ± 0.23	1.12
	1 × 10^4^	23.83 ± 0.15	0.63	23.91 ± 0.17	0.71
	1 × 10^3^	27.1 ± 0.08	0.30	27.18 ± 0.19	0.70
	1 × 10^2^	30.73 ± 0.08	0.26	30.78 ± 0.12	0.39
	Negative control	ND	ND	ND	ND

ND means no data.

### 3.6 Validation of the triplex real-time PCR assay using commercial singleplex real-time PCR kits

A comparison was conducted between triplex real-time PCR and commercial singleplex real-time PCR kits using thirty clinical samples. Supplementary Table S1 demonstrates that the triplex real-time PCR results align with those of the commercial singleplex real-time PCR, indicating its efficacy in concurrently detecting PRV, PTV1, and SS2.

### 3.7 Clinical implementation of the triplex real-time PCR

**Figure 3:**
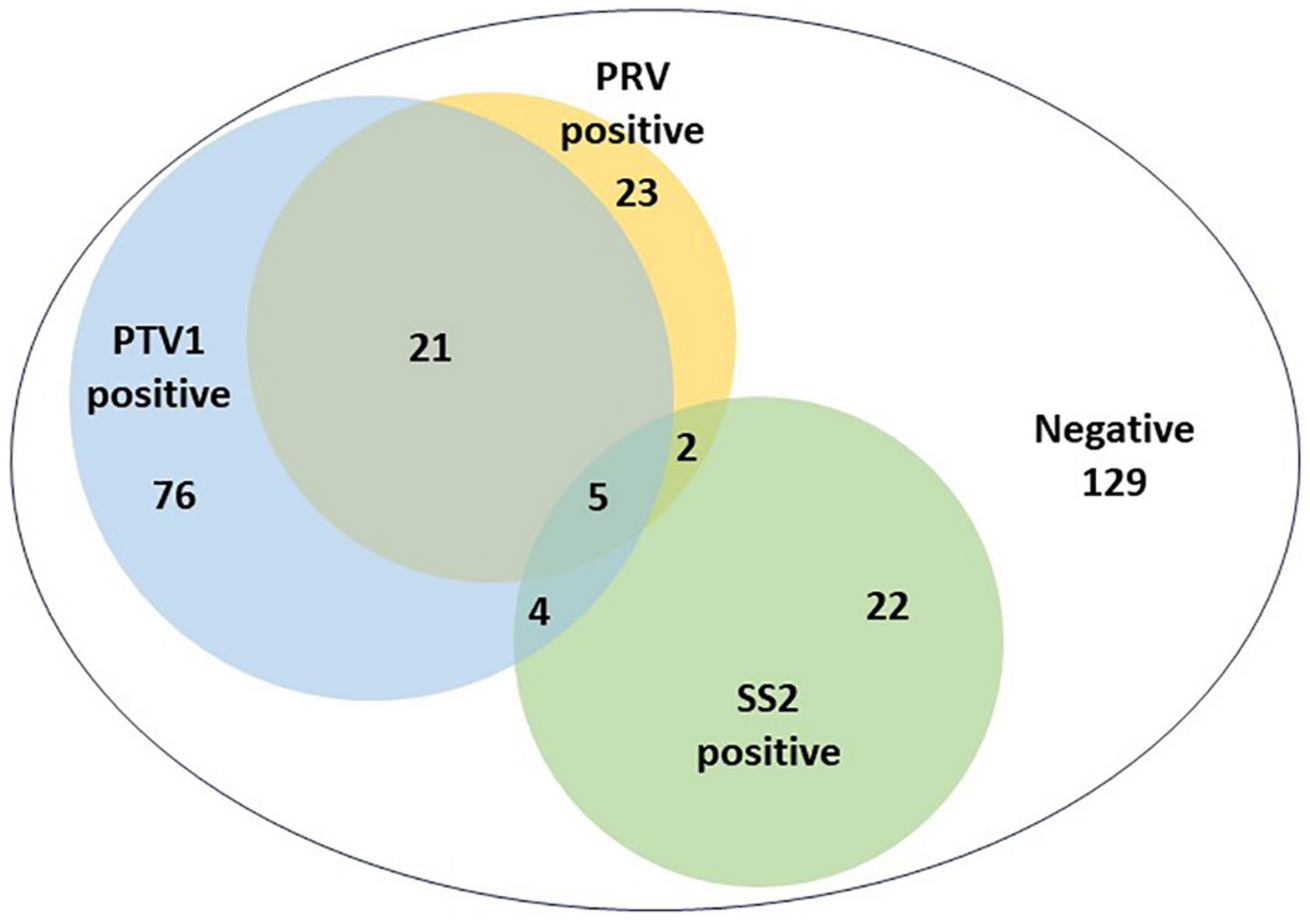
The detection results of clinical nervous samples using the triplex real-time PCR assay established in this study.

The developed triplex real-time PCR assay in this study was used to analyze 282 clinical samples of animals with nervous symptoms. Figure 3 illustrated the individual infection rates for PRV, PTV1, and SS2 as 8.16% (23 out of 282), 26.95% (76 out of 282), and 7.80% (22 out of 282), respectively. The co-infection rates were 7.45% for PRV + PTV1 (21/282), 0.71% for PRV + SS2 (2/282), and 1.42% for PTV1 + SS2 (4/282). The mixed infection rate for PRV + PTV1 + SS2 was 1.77% (5/282).

## 4 Discussion

Nervous system symptoms are frequently observed in clinical settings within the swine industry. The rapid and precise detection and identification of pathogens are essential for the prevention and control of infectious disease transmission (43, 44). Viral encephalitis in pig farms is caused by various pathogens such as PRV, PTV, Japanese encephalitis virus (JEV), porcine hemagglutinating encephalomyelitis virus (PHEV), atypical porcine pestivirus (APPV), porcine sapovirus (PSaV), porcine astroviruses (PoAstV), and porcine EMCV (45), with PRV being the most common in China. In contemporary intensive swine production systems, PTV can cause endemic outbreaks, and few herds or individual pigs remain free from PTV infection (21, 46). PTV1 is widely acknowledged as the primary etiological agent responsible for the majority of PTV cases characterized by severe clinical manifestations and high mortality within the PTV family (19). Historical outbreaks of PTV1 have been documented in the years 2000, 2004, 2009, and 2011 (12, 17, 47). Swine streptococcosis disease is designated as a Class II infectious disease in China (24), with up to 70% of SS isolates associated with systemic diseases in piglets belonging to serotype 2. Moreover, infections caused by SS2 are regarded as zoonotic (48, 49). There is an urgent requirement for a swift and accurate diagnostic technique that can concurrently distinguish between PRV, PTV1, and SS2.

This study involved the careful design of primers and probes aimed at the conserved regions of the PRV-gE, PTV1-VP1 and SS-CPS2J genes. Following extensive optimization, a robust triplex real-time PCR method was successfully developed, enabling the simultaneous detection of PRV, PTV1, and SS2 within a single amplification reaction. The method demonstrated exceptional sensitivity, with a detection threshold of 1 × 10^0^ copies/μL for the three target genes. The triplex real-time PCR demonstrated outstanding repeatability, with the CV of intra-assay between 0.05 and 0.63%, and the CV of inter-assay ranging from 0.04 to 1.12%. Thirty clinical samples of animals with neurological symptoms were used to assess and compare the effectiveness of commercial singleplex real-time PCR kits against our newly developed triplex real-time PCR method for detecting PRV, PTV1, and SS2. The study found the detection results were with full agreement between the two methods, indicating that the developed triplex real-time PCR assay could effectively replace commercial singleplex real-time PCR kits for differentiating PRV, PTV1, and SS2 simultaneously. To further validate the potential of the developed triplex real-time PCR assay for clinical application, 282 clinical samples were collected from animals with nervous symptoms to investigate the detection rates of these three pathogens. The detection rates for PTV1, PRV, and SS2 in these clinical samples were 37.59, 18.09, and 11.70%, respectively, aligning with previous findings that indicate the continued circulation of PTV1 in pig herds (50). The study found that 20.92% (32 of 153) of positive samples were co-infections, indicating a rising trend of multiple pathogen co-infections in large-scale, intensive swine production (Figure 3). Co-infections of PRV and PTV1 accounted for 13.73% (21/153) of positive samples, while PTV1 and SS2 co-infections were 2.62% (4/153) (Figure 3). Co-infections of PRV and SS2 were notably rare, occurring in only 1.31% (2 out of 153) of the samples (Figure 3). Importantly, 3.27% (5 out of 153) of the positive samples exhibited co-infections with PRV, PTV1, and SS2 (Figure 3). Despite the lack of data on PTV1, PRV, and SS2 co-infections, veterinarians should recognize that these three neurosystem-related diseases pose a critical threat to pig farms.

In conclusion, we have established a reliable triplex real-time PCR assay that distinguishes PRV, PTV1, and SS2. This assay demonstrates excellent specificity, sensitivity, and repeatability, effectively detecting clinical samples. Consequently, it serves as a valuable instrument for the rapid identification of these pathogens. The implementation of swift and accurate diagnostic measures, coupled with immediate quarantine and treatment protocols, may significantly contribute to the prevention and control of infectious disease dissemination.

## Data Availability

The original contributions presented in the study are included in the article/Supplementary material, further inquiries can be directed to the corresponding authors.

## Glossary

PRV: pseudorabies virus
PTV1: porcine teschovirus 1
SS2: *Streptococcus suis 2*
ED: edema disease
EMCV: encephalomyocarditis virus
SS: *Streptococcus suis*
HPS: *Haemophilus parasuis*
WOAH: World Organization for Animal Health
CPS: capsule polysaccharide antigens
ASFV: African swine fever virus
PRRSV: porcine reproductive and respiratory syndrome virus
PEDV: porcine epidemic diarrhea virus
PCV2: porcine circovirus type 2
PCV3: porcine circovirus type 3
CSFV: classical swine fever virus
TGEV: transmissible gastroenteritis virus
LOD: limit of detection
CV: coefficient of variation
PBS: phosphate buffer saline
JEV: Japanese encephalitis virus
PHEV: porcine hemagglutinating encephalomyelitis virus
APPV: atypical porcine pestivirus
PSaV: porcine sapovirus
PoAstV: porcine astroviruses
